# Metastasis‐associated protein 1 promotes epithelial‐mesenchymal transition in idiopathic pulmonary fibrosis by up‐regulating Snail expression

**DOI:** 10.1111/jcmm.15062

**Published:** 2020-03-18

**Authors:** Weibin Qian, Xinrui Cai, Qiuhai Qian, Wei Zhang, Li Tian

**Affiliations:** ^1^ Department of Lung Disease Affiliated Hospital of Shandong University of Traditional Chinese Medicine Jinan Shandong China; ^2^ Department of Traditional Chinese Medicine Shandong Academy of Occupational Health and Occupational Medicine Shandong First Medical University & Shandong Academy of Medical Sciences Jinan Shandong China; ^3^ Department of Endocrinology Affiliated Hospital of Shandong University of Traditional Chinese Medicine Jinan Shandong China; ^4^ First Clinical Medical College Shandong University of Traditional Chinese Medicine Jinan Shandong China

**Keywords:** astragaloside IV, epithelial‐mesenchymal transition, idiopathic pulmonary fibrosis, metastasis‐associated protein 1, snail

## Abstract

Idiopathic pulmonary fibrosis (IPF) is a progressive and usually fatal lung disease that lacking effective interventions. It is well known that aberrant activation of transforming growth factor‐beta1 (TGF‐β1) frequently promotes epithelial‐mesenchymal transition (EMT) in IPF. Metastasis‐associated gene 1 (MTA1) has identified as an oncogene in several human tumours, and aberrant MTA1 expression has been related to the EMT regulation. However, its expression and function in IPF remain largely unexplored. Using a combination of in vitro and in vivo studies, we found that MTA1 was significantly up‐regulated in bleomycin‐induced fibrosis rats and TGF‐β1‐treated alveolar type Ⅱ epithelial (RLE‐6TN) cells. Overexpression of MTA1 induced EMT of RLE‐6TN cells, as well as facilitates cell proliferation and migration. In contrast, knockdown of MTA1 reversed TGF‐β1‐induced EMT of RLE‐6TN cells. The pro‐fibrotic action of MTA1 was mediated by increasing Snail expression through up‐regulating Snail promoter activity. Moreover, inhibition of MTA1 effectively attenuated bleomycin‐induced fibrosis in rats. Additionally, we preliminarily found astragaloside IV (ASV), which was previously validated having inhibitory effects on TGF‐β1‐induced EMT, could inhibit MTA1 expression in TGF‐β1‐treated RLE‐6TN cells. These findings highlight the role of MTA1 in TGF‐β1‐mediated EMT that offer novel strategies for the prevention and treatment of IPF.

## 1 INTRODUCTION

Idiopathic pulmonary fibrosis (IPF) is a chronic, progressive inflammatory disease of the interstitial lung, and transformation of myofibroblast from fibroblast is a hallmark of IPF.^1^ During the development of IPF, myofibroblast not only expresses α‐smooth muscle actin (α‐SMA), but also produces extracellular matrix (ECM) production, such as type I collagen, leading to the IPF.^2^ Epithelial‐to‐mesenchymal transition (EMT) is highlighted as an important, possible mechanism of IPF. During EMT, the epithelial cells lose epithelial characteristics and acquired the mesenchymal phenotype with increased proliferative and migratory ability.^3^, ^4^, ^5^ It is well known that transforming growth factor‐beta1 (TGF‐β1), which is a pro‐fibrotic factor, has a pivotal role in inducing EMT.^6^ Therefore, novel therapeutic strategies should be focused on regulating TGF‐β1‐mediated EMT for effective management for IPF.

Metastasis‐associated protein 1 (MTA1) is a member of the MTA family (including MTA1, MTA2 and MTA3), which is served as the constituents of the Mi‐2/nucleosome remodelling and deacetylase protein complex.^7^ To date, MTA1 has been discovered to play indispensable roles in cell proliferation, tumorigenesis and metastasis, and it is known as a tumour inhibitor in many cancers.^8^, ^9^ However, the implication of MTA1 in IPF is undefined. Proteins of the MTA family have been identified as critical regulators of the EMT process, especially MTA1.^10^, ^11^ For instance, MTA1 enhances cell invasion and migration by inducing EMT in several types of cancers.^12^, ^13^, ^14^ We, therefore, have been suggested that MTA1 may involve in TGF‐β1‐indued EMT and play a potential role during the development of IPF.

Based on the above analysis, this study aimed to determine the expression of MTA1 in IPF and to investigate the regulation of MTA1 on EMT in bleomycin‐induced fibrosis in vivo, as well as TGF‐β1‐treated alveolar epithelial cells in vitro. On the other hand, we have previously demonstrated that astragaloside IV (ASV), the active substances of traditional Chinese medicinal plant astragalus membranaceus, inhibited TGF‐β1‐mediated EMT.^15^ Therefore, we also explored the regulation of ASV on MTA1 expression, to provide a potentially effective agent that targeting MTA1 in IPF.

## 2 MATERIALS AND METHODS

### 2.1 Cell culture and treatment

Alveolar type Ⅱ epithelial (RLE‐6TN) was obtained from ATCC (Rockville, Maryland, USA). The RLE‐6TN cells were cultured in 1640 medium (Hyclone) with 10% foetal bovine serum (FBS; Thermo Fisher Scientific), 1% penicillin/streptomycin (Beyotime) at 37°C with 5% CO_2_ in a humidified atmosphere. For inducing EMT, the complete medium was replaced with serum‐free medium (containing 0.1% FBS), in which the cells were incubated with recombinant TGF‐β1 (10 ng/mL, PeproTech).^15^

### 2.2 Plasmid construct and cell transfection

For MTA1 overexpression, the full‐length MTA1 cDNA was amplified and subcloned into pcDNA3.1. The empty pcDNA3.1 vector was served as the control. ShRNA targeting MTA1 (sh‐MTA1) was synthesized for MTA1 knockdown, and a scrambled shRNA (sh‐Scram) was synthesized for the negative control. The sequence used was presented in Table 1. In addition, siRNA oligo for smad3 and snail, as well as scrambled siRNA (si‐NC), was purchased from Genepharma. The sequence was listed in Table 1. The knockdown efficiency was determined by RT‐qPCR. For transfection, cells were cultured in six‐well plates until 70% confluence. Plasmid transfection was conducted by using Lipofectamine 2000 (Invitrogen).

**Table 1:** The sequence for plasmid and adeno‐associated virus in this study

Genes	5′‐3′ sequence
sh‐MTA1	GTACCGGAGACATCACCGACTTGTTAAACTCGAGTTTAACAAGTCGGTGATGTCTTTTTTTG
sh‐Scram	CCGGCAACAAGATGAAGAGCACCAACTCGAGTTGGTGCTCTTCATCTTGTTGTTTTTG
Si‐smad3	GCACTGACCATAAGAGCAA
Si‐NC	GTTGCGAAGATCGAGCAAA
Si‐snail	GCAGGATCTATCCCTGAAA
Si‐NC	GCGTGACGTTTAAACTATT
adeno‐associated virus‐sh‐MTA1	CCGGCCGCAGGATTGAAGAGCTTAACTCGAGTTAAGCTCTTCAATCCTGCGGTTTTTG
adeno‐associated virus‐sh‐Scram	CCGGCCGCAGTCTGAGACGGGTAGCTTACCTGATACTAGAATACTTATCCGGTTTTTG

### 2.3 Cell proliferation

Cell counting kit‐8 assay (MedChemExpress) was performed to evaluate cell proliferation. RLE‐6TN cells (5000 per well) were plated into 96‐well plates. After transfected with sh‐MTA1 or sh‐Scram, the CCK‑8 reagent was added to each well (dilution, 1:10) and cells were incubated for 2 hours at 37°C. The cell viability was measured at 450 nm using a microplate reader.

### 2.4 Transwell assay

Transwell chambers (8 μm pore size membranes) were used for cell migration assay. After transfection, 3 × 10^5^ RLE‐6TN cells were plated on the top chamber and incubated in medium without FBS. The medium with 10% FBS was placed in the lower chamber. After 24 hours in the incubator at 37℃, non‐migrated cells were gently removed and cells on the lower chamber were fixed with 0.5% crystal violet. The number of migrated cells was calculated under the Nikon Eclipse Ti microscopy.

### 2.5 Real‐time quantitative PCR

Total RNA from pulmonary tissues or cells was isolated by TRIzol (Invitrogen). cDNA was obtained from 2 μg of total RNA by reverse transcription by using PrimeScript reagent Kit (Takara). RT‐qPCR was performed on a LightCycler96 Real‐Time PCR System (Roche, Basel, Switzerland) with the SYBR Green PCR Master Mix (Takara). GAPDH was used as internal controls. The relative expression of target genes was calculated by the 2^−ΔΔCt^ method.^16^ The primers were shown in Table 2.

**Table 2:** The forward and reverse primers for real‐time PCR

Genes		5′‐3′ primer sequence
MTA1	Forward	AGGGAATGCCAGTCCGAAAC
	Reverse	TGTAGAATGAATCAGGGAAAGC
Col1α	Forward	TGTGCGATGACGTGATCTGTGA
	Reverse	CTTGGTCGGTGGGTGACTCTG
Col3α	Forward	GCTGAAGGGCAGGGAACAAC
	Reverse	GTGAAGCAGGGTGAGAAGAAA
CTGF	Forward	AGACCCAACTATGATTAGAGCCA
	Reverse	CCGTCGGTACATACTCCACA
GAPDH	Forward	GGTGCTGAGTATGTCGTGGAG
	Reverse	ACAGTCTTCTGAGTGGCAGTGAT

### 2.6 Western blot analysis

Total protein was isolated from pulmonary tissues or cells by using 1% RIPA Buffer (Beyotime, Jiangsu, China), and 30 μg of denatured protein extracts were resolved by 12% sodium dodecyl sulphate‐polyacrylamide gel electrophoresis. The proteins were transferred to polyvinylidene difluoride membranes (Millipore), which were blocked in 5% fat‐free milk in 0.1% tween‐20 tris‐buffered saline for 1 hour at room temperature. The blots were subsequently incubated with antibodies against MTA1 (ab71153, Abcam), MTA2 (ab8106, Abcam), MTA3 (ab87275, Abcam), α‐SMA (ab203829, Abcam), Collagen1 (ab32503, Abcam), Snail (ab180714, Abcam), phospho‐smad3 (cat.9520, CST) and smad3 (cat.9523, CST) at 4°C overnight. Next membranes were incubated with horseradish peroxidase‐conjugated secondary antibody (ab6721, Abcam) for 1 hour at 24°C. All blots were imaged using an enhanced chemiluminescence detection kit (Beyotime). β‐actin (sc‐70319, Santa Cruz) was considered as the internal control.

### 2.7 Luciferase reporter assays

Luciferase reporter assay was performed as previously published. In brief, the snail gene promoter region, −350 to + 84 bps relative to the transcription start site, was cloned into pGL3 vector (Promega). RLE‐6TN cells (1 × 10^4^/well) were co‐transfected with pLG3‐Snail internal control plasmid (expressing Renilla luciferase), along with pcDNA3.1 MTA1 or pcDNA3.1 vector by Lipofectamine 2000 (Invitrogen). The cell lysate was collected after 24 h of transfection, and the luciferase activities were measured by using a Dual‐Luciferase Reporter Assay kit (Promega).

### 2.8 Pulmonary fibrosis model

A total of 40 rats were randomly assigned to 4 groups: control group (received a single intratracheal instillation of 50 mL saline), IPF group (received a single intratracheal instillation of 50 mL saline containing 5 mg/kg bleomycin), sh‐MTA1 group (MTA1 knockdown adeno‐associated virus was intraperitoneally injected to knockdown MTA1) and sh‐Scram group (adeno‐associated virus containing Scrambled shRNA was intraperitoneally injected). All rats were sacrificed after treatment for 28 days, and the lung tissues were collected followed by fixation and embedding. Procedures involving animals and their care were conducted following a protocol approved by the Ethics Committee of the Affiliated Hospital of Shandong University of Traditional Chinese Medicine.

### 2.9 Histology and immunohistochemistry

The paraffin‐embedded tissues were cut into 4‐μm thick sections. The sections stained with haematoxylin and eosin (H&E) staining and Masson's trichrome staining following the manufacturers’ instructions. For immunohistochemistry, sodium citrate was used for antigen retrieval, and 0.3% hydrogen peroxide was used to block endogenous peroxidase. The slides were then incubated with the anti‐MTA1 antibody (ab87275) at 1:300 dilution overnight at 4℃. The second day, sections were incubated with horseradish peroxidase‐conjugated goat anti‐rabbit IgG. After that, the slides were counterstained with haematoxylin, followed by dehydration in graded alcohols and xylene. Immunopositive cells were quantified using ImageJ software.

### 2.10 Statistical analysis

Data analysis was performed using SPSS version 22.0 software (SPSS Inc). The Student's t test or one‐way analysis of variance (ANOVA) was used for comparison. Error bars represent the standard deviation of the mean (SD), as indicated. Differences with *P*‐values of less than .05 were considered as statistically significant.

## 3 RESULTS

### 3.1 MTA1 is increased in bleomycin‐induced fibrosis rats and TGF‐β1‐treated RLE‐6TN cells

**Figure 1:**
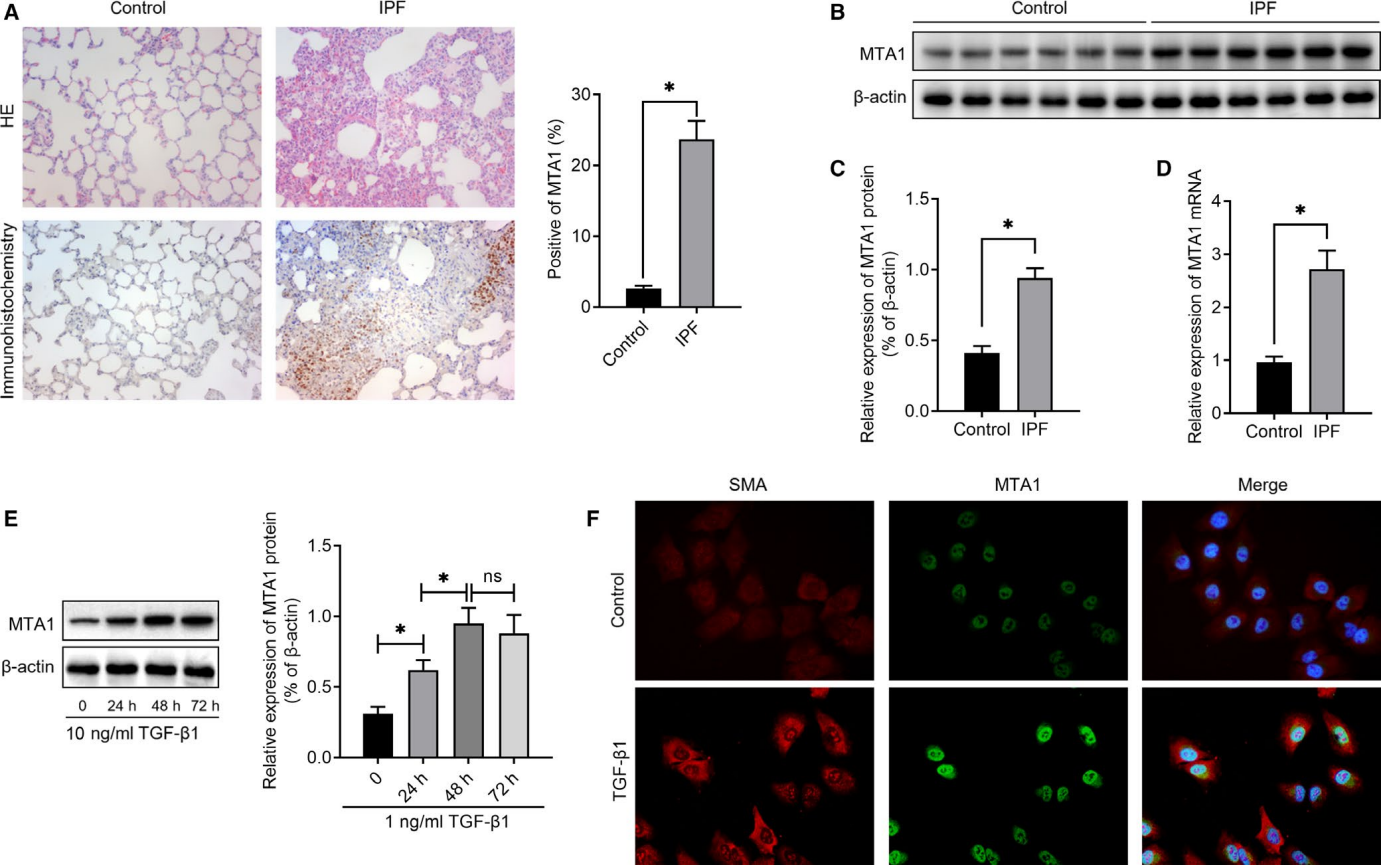
MTA1 is up‐regulated in bleomycin‐induced rats and TGF‐β1‐treated RLE‐6TN cells. A, Haematoxylin and eosin (H&E) staining was used to show the histologic change in bleomycin‐induced IPF; the expression of MTA1 was analysed by immunohistochemistry, and positive rate of MTA1 was presented as a histogram. B‐C, Expression of MTA1 protein in pulmonary tissues from control or bleomycin‐induced rats was analysed by Western blot analysis. D, RT‐qPCR was performed to detect the expression of MTA1 mRNA in pulmonary tissues. E, Western blot analysis was used to determine the change of MTA1 expression in RLE‐6TN cells upon 10 ng/mL TGF‐β1 treatment for various time. F, Immunofluorescence was used to detect the expression of α‐SMA and MTA1 in RLE‐6TN cells with or without 10 ng/mL TGF‐β1 treatment for 48 h. *P < .01 vs control

Firstly, H&E staining was used to validate that bleomycin could induce pulmonary fibrosis in our study. To determine the profile of MTA1 in IPF, immunohistochemistry was performed and the results in Figure 1A showed that compared with the control one, the expression levels of MTA1 were obviously up‐regulated in the rats with pulmonary fibrosis. To exactly measure MTA1 levels, we performed Western blot and RT‐qPCR analysis and the results revealed that MTA1 was significantly increased in bleomycin‐induced fibrosis, both at protein levels (Figure 1B and 1C) and mRNA levels (Figure 1D). In addition, to investigate the change of MTA1 upon TGF‐β treatment, as well as the cellular localization of MTA1 in alveolar cells, we treated RLE‐6TN cells with 10 ng/mL TGF‐β1. As displayed in Figure 1E, the addition of TGF‐β1 increased MTA1 levels significantly in a time‐dependent manner and the levels of MTA1 reached the peak at 48 hours following TGF‐β1 treatment. Immunofluorescence analysis then illustrated that MTA1 was mostly located in the nucleus of alveolar cells, and slightly in the cytoplasm. Accordingly, TGF‐β1 treatment increased the levels of α‐SMA, which promote us to investigate the potential role of MTA1 in regulating EMT (Figure 1F).

### 3.2 Overexpression of MTA1 induces EMT of RLE‐6TN cells

**Figure 2:**
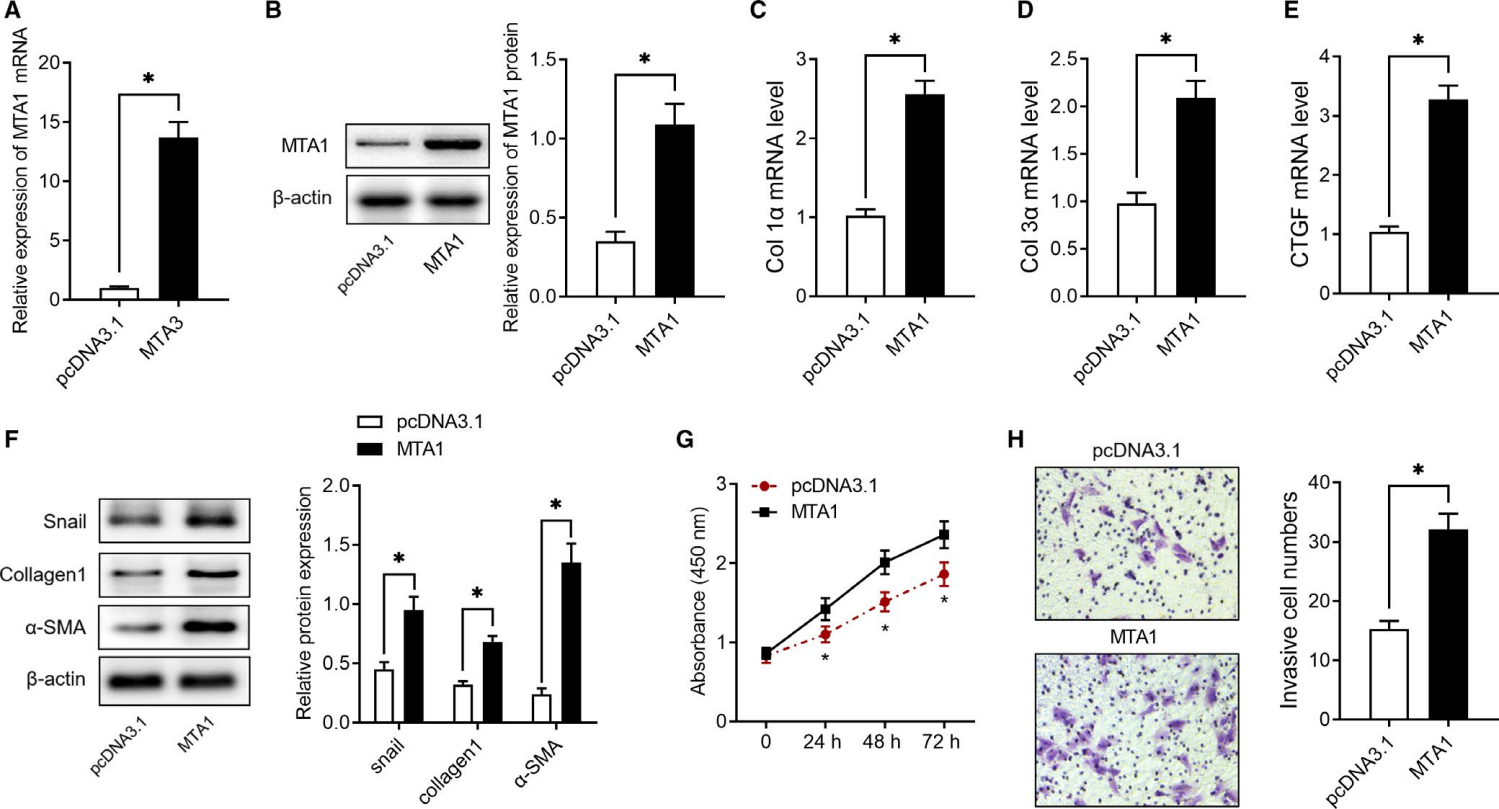
Overexpression of MTA1 results in fibrogenesis in RLE‐6TN cells. A‐B, Overexpression of MTA1 was achieved by transfected with pcDNA3.1 carrying MTA1 gene, and the results were validated by RT‐qPCR and Western blot analysis. C‐E, Relative expression of Col 1α, Col 3α and CTGF in RLE‐6TN cells with or without overexpression of MTA1, as detected by RT‐qPCR. F, Relative expression of Snail, collagen1 and α‐SMA in RLE‐6TN cells with or without overexpression of MTA1, as detected by Western blot analysis. G, Cell proliferation of RLE‐6TN cells was evaluated by CCK‐8 assays after overexpression of MTA1. H, Cell migration was evaluated by Transwell assay after overexpression of MTA1. *P < .05 vs. pcDNA3.1

To evaluate the function of MTA1 in IPF, we firstly overexpressed MTA1 in vitro. As shown in Figure 2A,B, transfection with pcDNA3.1 carrying MTA1 sequencing significantly increased MTA1 expression both at mRNA and protein levels, as evidenced by RT‐qPCR and Western blot analysis. Subsequent experiments found overexpression of MTA1 notably up‐regulated extracellular matrix (ECM)‐related genes, including Col1α, Col3α and CTGF (Figure 2C‐E). Consistently, overexpression of MTA1 increased extracellular matrix‐related proteins, including Snail, collagen1 and α‐SMA (Figure 2F). Furthermore, the results of the CCK‐8 assay showed that MTA1 overexpressing promoted cell viability (Figure 2G) and Transwell assay showed that its overexpression facilitated cell migration ability (Figure 2H).

### 3.3 Inhibition of MTA1 reverses TGF‐β1‐induced EMT by regulating snail

**Figure 3:**
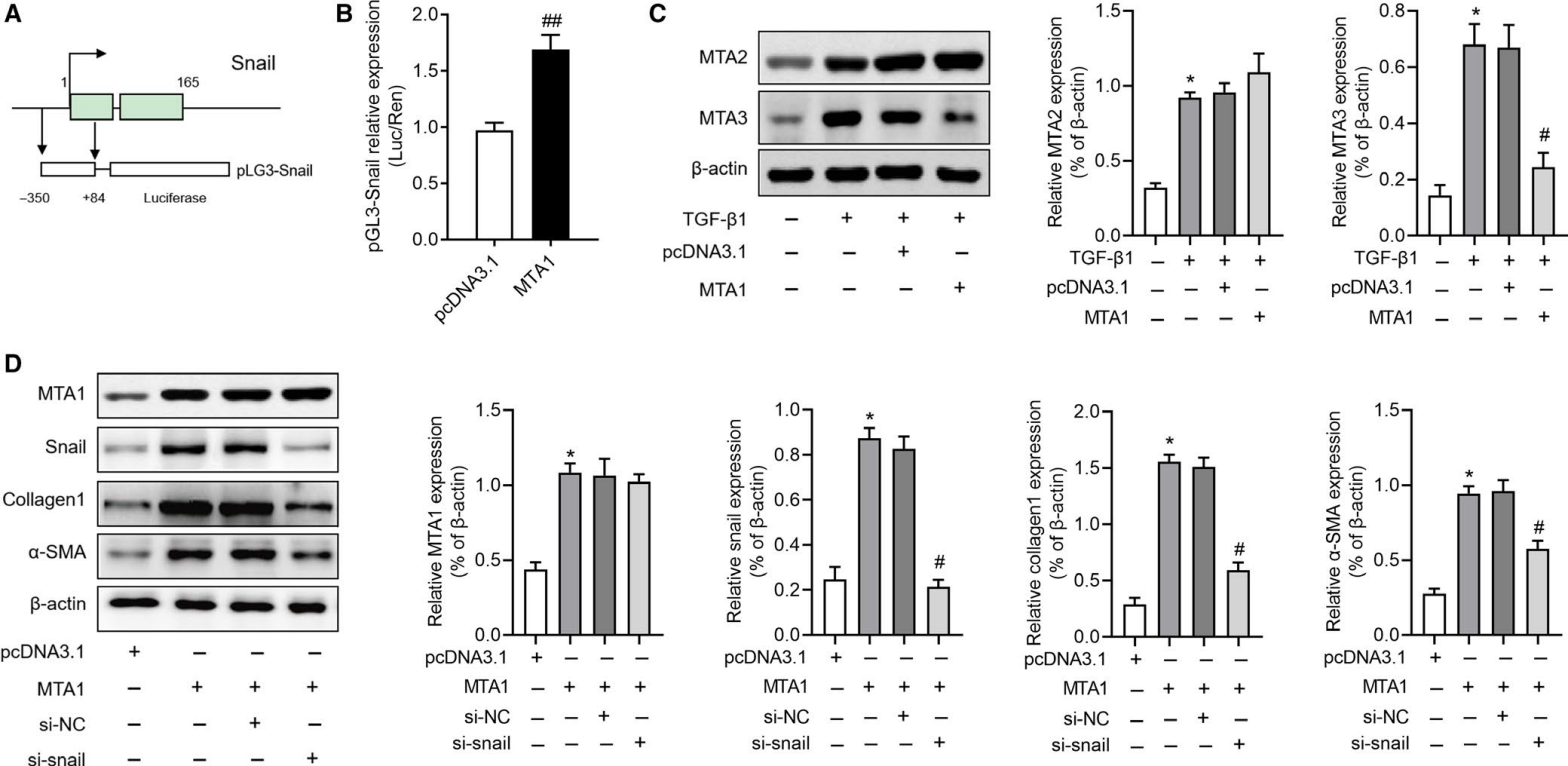
MTA1 regulates EMT of RLE‐6TN cells by targeting snail. A, The schematic diagrams of pGL3‐snail used in a luciferase reporter assay. B, Overexpression of MTA1 via plasmid transfection resulted in significant up‐regulation of snail promoter activity in RLE‐6TN cells. C, Relative expression of MTA2 and MTA3 protein in RLE‐6TN cells transfected with pcDNA3.1 or MTA1 under TGF‐β1 treatment. D, Relative expression of MTA1, snail and EMT‐related proteins, including collagen1 and α‐SMA, was determined in RLE‐6TN cells with MTA1 overexpression and/or snail silencing. * *P* < .05 vs control; **P < .05 vs TGF‐β1 + pcDNA3.1; ^#^ *P* < .05 vs MTA1 + si‐NC; ^##^ *P* < .05 vs pcDNA3.1

To date, the mechanism underlying the regulation of MTA1 on EMT is unclear, we therefore further investigate the regulation of MTA1 on Snail expression, an important factor in regulating EMT. Luciferase reporter assay in our study showed that after overexpression of MTA1, the transcriptional activity of the Snail promoter was significantly up‐regulated (Figure 3A,B). A previous study indicated that up‐regulation of MTA1 could result in the repression of MTA3 expression and up‐regulated of Snail,^7^ we then investigated whether MTA1 affected MTA2 and MTA3 in RLE‐6TN cells. As shown in Figure 3C, TGF‐β1 both up‐regulated MTA2 and MTA3 expression. Overexpression of MTA1 had no effects on MTA2 expression but inhibited MTA3 expression in our study. Moreover, we knockdown snail in MTA1 overexpressed RLE‐6TN cells and further found that Snail silencing could partly reverse MTA1 overexpression induced collagen1 and α‐SMA expression but showed no changes on MTA1 expression (Figure 3D). These results indicated that MTA1 regulates EMT in a Snail‐dependent way.

**Figure 4:**
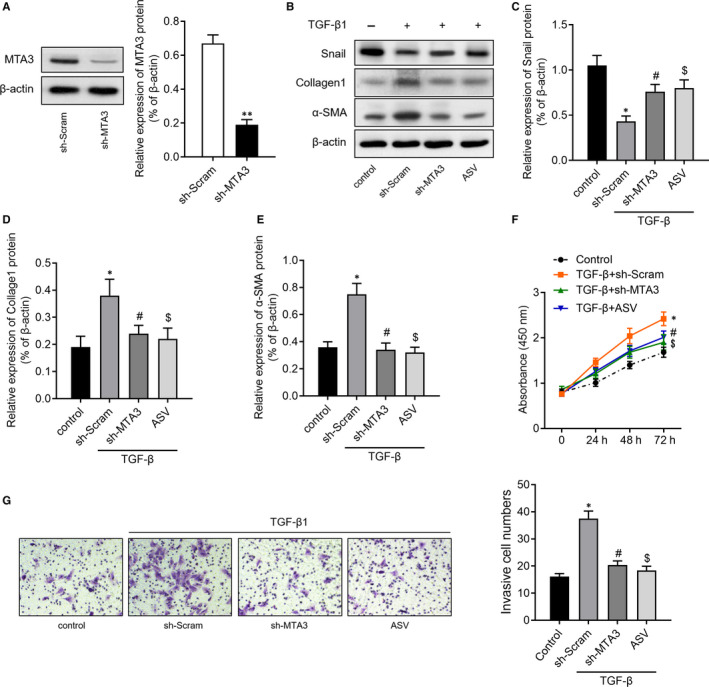
Inhibition of MTA1 or astragaloside IV (ASV) treatment reverses TGF‐β1‐induced EMT. A, Relative expression of MTA1 protein in RLE‐6TN cells transfected with sh‐MTA1 or control vector (sh‐Scram). B‐E, Relative expression of EMT‐related proteins, including snail, collagen1 and α‐SMA, was determined in RLE‐6TN cells with MTA1 silencing or ASV treatment. F, CCK‐8 assay was performed to assess the cell proliferation of RLE‐6TN cells transfected with sh‐MTA1, sh‐Scram or ASV treatment. G, Transwell assay was performed to detect cell migration of RLE‐6TN cells with MTA1 silencing or ASV treatment. *P < .05 vs control; ^#^ *P* < .05 vs TGF‐β + sh‐Scram

Thereafter, we knockdown MTA1 by transfecting RLE‐6TN cells with sh‐MTA1 (Figure 4A). Moreover, we also treated RLE‐6TN cells with 100 μg/mL astragaloside IV (ASV), which was previously validated having inhibitory effects on TGF‐β1‐induced EMT.^15^ The results of Western blot then revealed that TGF‐β1 treatment increased the expression of Snail, collagen1 and α‐SMA. However, inhibition of MTA1 or ASV treatment could partly reverse the change of Snail, collagen1 and α‐SMA induced by TGF‐β1 (Figure 4B‐E). CCK‐8 assay (Figure 4F) and Transwell assay (Figure 4G) revealed that inhibition of MTA1 or ASV treatment significantly abolished TGF‐β1‐mediated cell proliferation and migration of RLE‐6TN cells.

### 3.4 ASV suppresses MTA1 expression by regulating TGF‐β1/smad3 signalling

**Figure 5:**
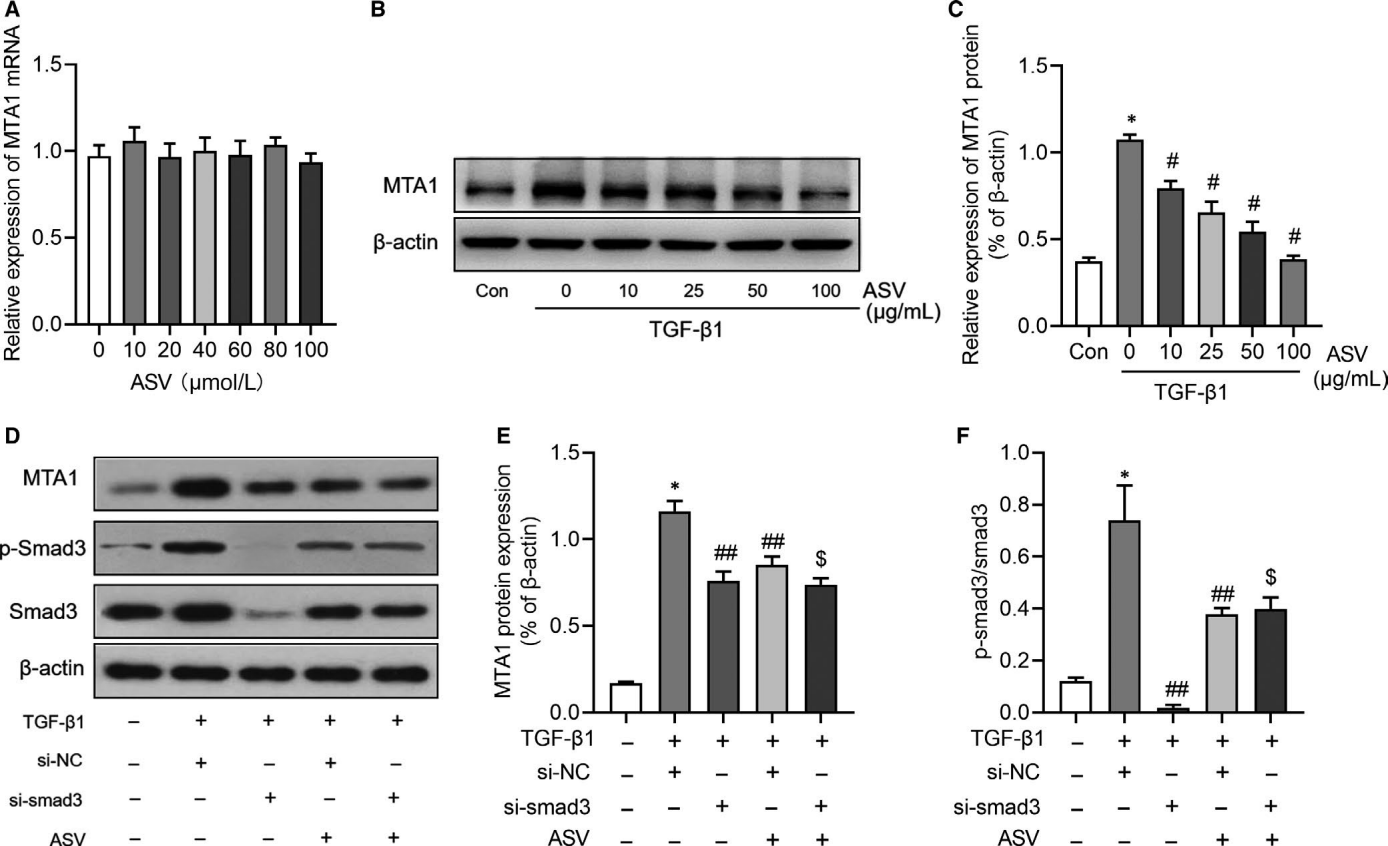
Astragaloside IV (ASV) suppresses MTA1 expression via TGF‐β1/smad3 signalling. A, Relative expression of MTA1 mRNA in RLE‐6TN cells with different concentrations of ASV, as detected by RT‐qPCR. B‐C, Relative expression of MTA1 in TGF‐β1‐treated RLE‐6TN cells with different concentrations of ASV, as detected by Western blot analysis. D‐F, Relative expression of MTA1, p‐smad3 and smad3 protein in TGF‐β1 or ASV‐treated RLE‐6TN cells with or without knockdown of smad3. *P < .05 vs control; ^#^ *P* < .05 vs TGF‐β1; ^#^ *P* < .05 vs TGF‐β1 + si‐NC; ^$^ *P* < .05 vs TGF‐β1 + si‐NC + ASV

As depicted in Figure 5A, a series of concentrations of ASV had no effects on MTA1 mRNA expression. However, ASV could inhibit MTA1 protein expression in a concentration‐dependent manner in TGF‐β1‐treated RLE‐6TN cells (Figure 5B,C). We then explored how ASV influenced the expression of MTA1. As a previous study had shown that ASV effectively attenuated the progression of IPF by regulating TGF‐β1/smad signalling,^15^, ^17^ we therefore tested whether ASV regulated MTA1 through TGF‐β1 signalling. Interesting, the expression of MTA1, as well as the ratio of p‐smad3/smad3, was suppressed in si‐smad3 group when compared with that in si‐NC group. Besides, knockdown of smad3 further decreased MTA1 expression in ASV‐treated cells (Figure 5D‐F), indicating that TGF‐β1/smad3 signalling may be involved in the regulation of ASV on MTA1 in alveolar cells.

### 3.5 Inhibition of MTA1 attenuates bleomycin‐induced fibrosis in rats

**Figure 6:**
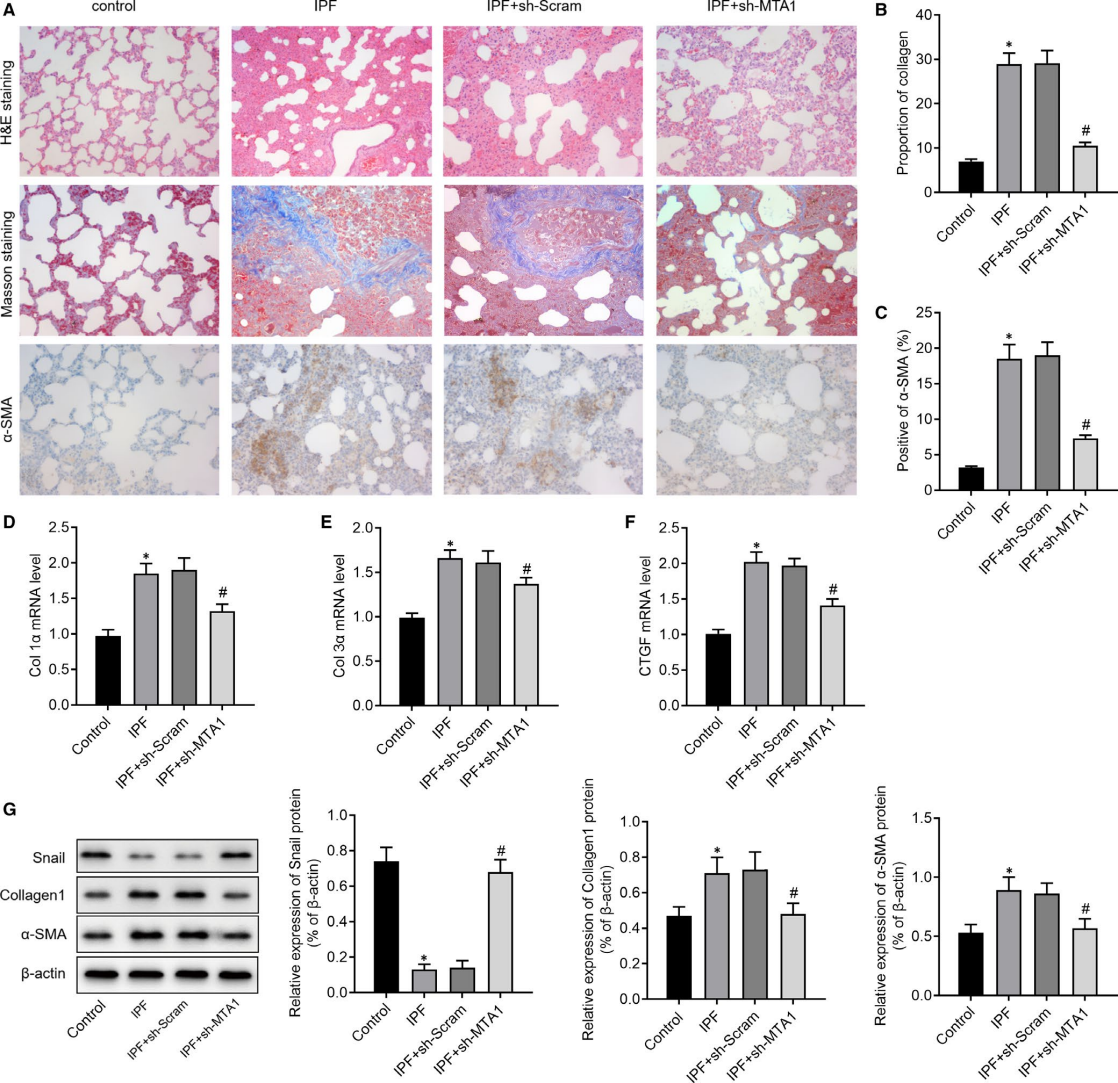
Inhibition of MTA1 attenuates bleomycin‐induced IPF in rats. A‐B, Haematoxylin and eosin (H&E) and Masson staining were performed to show the histologic change and alteration of collagen accumulation in IPF rats with or without MTA1 silencing. (A and C) Immunohistochemistry showed the change of the positive rate of α‐SMA in IPF rats with or without MTA1 silencing. D‐F, Relative expression of Col 1α, Col 3α and CTGF in pulmonary tissues with or without MTA1 silencing was detected by RT‐qPCR. G, Relative expression of Snail, collagen1 and α‐SMA in pulmonary tissues with or without MTA1 silencing was detected by Western blot analysis. *P < .05 vs control; ^#^ *P* < .05 vs IPF + sh‐Scram

Subsequently, we investigated the role of MTA1 in vivo. H&E, Masson's trichrome staining and immunohistochemistry were performed. The results in Figure 6A‐C showed that inhibition of MTA1 attenuated bleomycin‐induced fibrosis, as evidenced by the reduced lymphocyte infiltration and amounts of collagen. Moreover, we found that inhibition of MTA1 in bleomycin‐treated rats pronouncedly decreased the expression of myofibroblast‐expressed genes, including Col1α, Col3α and CTGF (Figure 6D‐F). Western blot analysis of pulmonary tissues showed increased ECM‐related proteins, Snail, collagen1 and α‐SMA in IPF rats, while knockdown of MTA1 increased snail and reduced collagen1 and α‐SMA (Figure 6G).

## 4 DISCUSSION

In the present study, we characterized the pro‐fibrotic property of MTA1 in IPF and elucidated the molecular mechanism underlying the involvement of MTA1 in TGF‐β1‐mediated fibroblast activation. We demonstrated that MTA1 was significantly up‐regulated in IPF. Overexpression of MTA1 promotes lung fibrosis by trigging EMT of alveolar epithelial cells. In contrast, inhibition of MTA1 inhibited TGF‐β1‐induced EMT by transcriptional activation of Snail expression, therefore to attenuate bleomycin‐induced IPF in rats. These results indicated the regulatory role of MTA1 in TGF‐β1‐induced EMT, providing the evidence about inhibition of MTA1 as a promising therapeutic strategy for IPF.

It has been suggested that the MTA1 gene is a potential candidate oncogene in several types of human malignancies.^18^ MTA1 protein interacts with histone deacetylase to form a nucleosome remodelling histone deacetylase complex, which could regulate oncogenesis and angiogenesis in a variety of cancers.^19^, ^20^, ^21^ MTA1 promotes the progression of cancer by inducing EMT, which functions as a central mediating process that contributes to IPF.^22^, ^23^ Up to now, however, the expression changes of MTA1 and its biological functions and mechanisms in the pathogenic process of IPF are unknown. In this study, we for the first time showed that MTA1 was up‐regulated in the lung tissues from bleomycin‐treated rats and TGF‐β1‐treated RLE‐6TN cells. We thus propose that the up‐regulated expression of MTA1, which induced EMT, could also participate in the development of IPF. As expected, we found overexpression of MTA1 could promote fibroblast activation as evidenced by up‐regulated myofibroblast‐related genes, including Col 1α, Col 3α and CTGF. In addition, MTA1 overexpression induced EMT, which was evidenced by the up‐regulated the expression of Snail, collagen1 and α‐SMA.^24^ In contrast, inhibition of MTA1 reversed TGF‐β1‐induced EMT and bleomycin‐induced fibrosis.

Although plenty of studies have validated the role of MTA1 in regulating EMT, its exact mechanisms are still needed to be revealed. We noticed that MTA3 could repress the expression of Snail and inhibition of MTA3 resulted in an increase of Snail promoter activities.^25^ Considering that MTA1 could negatively interfere with the transactivation function of MTA3,^10^ we then explored whether MTA1 regulates Snail expression. And interesting, we found that MTA1 overexpression leading to a decrease in MTA3 expression and a significant increase in snail promoter activities, as well as Snail protein expression. Further, inhibition of Snail reversed MTA1 overexpression induced EMT in RLE‐6TN cells. Conversely, inhibition of MTA1 partly abolished TGF‐β1‐induced Snail expression. Taken together, our data indicated that MTA1 induced EMT by promoting Snail expression.

On the other hand, we had previously focused on finding effective approaches to inhibit the progression of IPF and found ASV, a monomeric compound isolated from astragalus, could effectively alleviate IPF by inhibiting TGF‐β1‐induced EMT.^15^ However, whether ASV could influence MTA1 expression and its potential mechanism in alveolar cells upon TGF‐β1 treatment is unknown. This study for the first time demonstrated that addition of ASV could suppress MTA1 expression up‐regulated by TGF‐β1 through regulating TGF‐β1/smad3 signalling. Consistent with our previous study,^15^ a study by Li et al also identified ASV could effectively regulate TGF‐β1 signalling to alleviate IPF.^17^ Considering that MTA1 status is a determinant of TGF‐β1‐induced EMT phenotypes,^26^ it is reasonably summing up that ASV regulated MTA1 expression in a TGF‐β1‐dependent way. Although how TGF‐β1/smad3 regulates MTA1 expression still needs to be clarified, the data in this study further a new potential molecular mechanism of the protective of ASV on IPF.

Overall, our study demonstrated that MTA1 inhibition could reverse the formation of the pulmonary EMT process by suppressing the expression of Snail. Thus, MTA1 inhibition represents a promising therapeutic target for patients with IPF that deserves further study and evaluation.

## CONFLICTS OF INTEREST

The authors confirm that there are no conflicts of interest.

## AUTHOR CONTRIBUTIONS

XC and QQ designed the study and performed the statistical analysis; QQ, WQ and WZ did most of the animal experiments; WQ, XC and LT performed the in vitro experiments; WQ, XC and WZ wrote the manuscript.

## Data Availability

All data generated or analysed during the study are included in this published article.
